# Infection prevention strategies are highly protective in COVID-19 units while main risks to healthcare professionals come from coworkers and the community

**DOI:** 10.1186/s13756-021-01031-5

**Published:** 2021-11-22

**Authors:** Shruti K. Gohil, Kathleen A. Quan, Keith M. Madey, Suzanne King-Adelsohn, Tom Tjoa, Delia Tifrea, Bridgit O. Crews, Edwin S. Monuki, Saahir Khan, Sebastian D. Schubl, Cassiana E. Bittencourt, Neil Detweiler, Wayne Chang, Lynn Willis, Usme Khusbu, Antonella Saturno, Sherif A. Rezk, Cesar Figueroa, Aarti Jain, Rafael Assis, Philip Felgner, Robert Edwards, Lanny Hsieh, Donald Forthal, William C. Wilson, Michael J. Stamos, Susan S. Huang

**Affiliations:** 1 Epidemiology and Infection Prevention Program, Irvine Health (UC Irvine Health), University of California, Irvine, USA; 2 Division of Infectious Diseases, Department of Medicine, Irvine School of Medicine, University of California, 100 Theory, Suite 120, Irvine, CA 92617 USA; 3 Pathology and Laboratory Medicine, UCI Health, Newport Beach, USA; 4 Division of Infectious Diseases, Keck School of Medicine, University of Southern California, Los Angeles, USA; 5 Department of Surgery, Irvine School of Medicine, University of California, Irvine, USA; 6 Division of Occupational and Environmental Medicine, Irvine School of Medicine, University of California, Irvine, USA; 7 Department of Physiology and Biophysics, University of California, Irvine, USA; 8 Chief Medical Officer, UC Irvine Health, Orange, USA

**Keywords:** COVID-19 seroprevalence, Healthcare worker COVID-19 exposure, COVID-19 outbreaks, Severe acute respiratory syndrome coronavirus-2, Healthcare professional COVID-19 exposure

## Abstract

**Background:**

Early evaluations of healthcare professional (HCP) COVID-19 risk occurred during insufficient personal protective equipment and disproportionate testing, contributing to perceptions of high patient-care related HCP risk. We evaluated HCP COVID-19 seropositivity after accounting for community factors and coworker outbreaks.

**Methods:**

Prior to universal masking, we conducted a single-center retrospective cohort plus cross-sectional study. All HCP (1) seen by Occupational Health for COVID-like symptoms (regardless of test result) or assigned to (2) dedicated COVID-19 units, (3) units with a COVID-19 HCP outbreak, or (4) control units from 01/01/2020 to 04/15/2020 were offered serologic testing by an FDA-authorized assay plus a research assay against 67 respiratory viruses, including 11 SARS-CoV-2 antigens. Multivariable models assessed the association of demographics, job role, comorbidities, care of a COVID-19 patient, and geocoded socioeconomic status with positive serology.

**Results:**

Of 654 participants, 87 (13.3%) were seropositive; among these 60.8% (N = 52) had never cared for a COVID-19 patient. Being male (OR 1.79, CI 1.05–3.04, *p* = 0.03), working in a unit with a HCP-outbreak unit (OR 2.21, CI 1.28–3.81, *p* < 0.01), living in a community with low owner-occupied housing (OR = 1.63, CI = 1.00–2.64, *p* = 0.05), and ethnically Latino (OR 2.10, CI 1.12–3.96, *p* = 0.02) were positively-associated with COVID-19 seropositivity, while working in dedicated COVID-19 units was negatively-associated (OR 0.53, CI = 0.30–0.94, *p* = 0.03). The research assay identified 25 additional seropositive individuals (78 [12%] vs. 53 [8%], *p* < 0.01).

**Conclusions:**

Prior to universal masking, HCP COVID-19 risk was dominated by workplace and community exposures while working in a dedicated COVID-19 unit was protective, suggesting that infection prevention protocols prevent patient-to-HCP transmission.

**Article summary:**

Prior to universal masking, HCP COVID-19 risk was dominated by workplace and community exposures while working in a dedicated COVID-19 unit was protective, suggesting that infection prevention protocols prevent patient-to-HCP transmission.

**Supplementary Information:**

The online version contains supplementary material available at 10.1186/s13756-021-01031-5.

## Introduction

Exposure to transmissible diseases is a known occupational hazard for healthcare professionals (HCPs), which warrants robust infection prevention protocols. Early reports from China demonstrated HCP COVID-19 infection rates as high as 44% and subsequent large-scale studies in early 2020 showed a 12-fold higher COVID-19 test-positivity rate among HCPs compared to the community, leading the healthcare facilities to adopt robust measures to protect against patient exposure to COVID-19 [1–5]. Though evidence is beginning to emerge that COVID-19 seroprevalence among HCPs mirrors communities in which they live [6–9], HCP perception is that their highest risk is during patient care,calling into question the effectiveness of infection prevention strategies deployed to protect them [10, 11].

Many currently available studies assess COVID-19 incidence or prevalence among randomly sampled HCPs and infer occupational risk by defining exposure broadly as having worked in healthcare or in a COVID-19 patient unit without addressing ongoing clusters/outbreaks related to coworkers working while ill or community exposures [2, 4, 5, 7–9]. HCP COVID-19 prevalence may also be exaggerated by testing bias from occupational health screening and ready access to tests [2]. Finally, HCPs are essential workers that require in-person activity, increasing the overall number of both community and work-related interactions within a given day. Epidemiologic studies are needed that assess the added risk borne by HCPs due to COVID-19 patient care compared to exposures from coworker in the healthcare setting and the communities where they live.

In this study, we evaluated whether HCP roles, documented COVID-19 patient care, coworker exposures, and geocoded community characteristics were associated with the likelihood of polymerase chain reaction (PCR) or serology positive COVID-19, enriching for those presenting to Occupational Health (OH) with symptoms, those working in COVID-19-designated care units, those on units with COVID-19 HCP outbreaks, and control non-COVID-19 designated units without HCP outbreaks. Our objective was to fully characterize HCP exposure risk factors retrospectively, after outbreak investigations, to identify any previously unidentified COVID-19 positive HCPs using serologic testing.

## Methods

### Study design and population

We conducted a retrospective cohort study combined with a cross-sectional seroprevalence survey of HCP at an academic medical center in Orange County, California between January 1, 2020-April 15, 2020. During this time, the first cases of COVID were identified (cumulative county-wide cases by April 15, 2020 were 1,635), testing availability was limited and largely relegated to hospitalized patients, and vaccines had not yet become available. All patients and HCPs underwent symptom or close-contact exposure screening upon entry into facility and daily. If any of 11 Centers for Disease Control & Prevention (CDC) defined symptom or close contact exposure screens were positive then patients were pre-emptively placed into COVID-19 precautions, HCPs were not allowed on premises until evaluated by Occupational Health, and all were tested for SARS-CoV-2 by PCR (see Data Collection for details). Universal masking was not yet in place and COVID-19 PPE (droplet masks, face shields, gowns and gloves) was used for patients suspected or confirmed to have COVID-19. To enrich for COVID-19 cases among HCPs, we included: all HCP assigned to designated COVID-19 units (3), hospital units that experienced a COVID-19 HCP outbreak, defined as 2 or more HCP with epidemiologically linked infections (3), and matched control units not designated for COVID-19 care and without an active HCP outbreak (3) during the study period [10]. HCPs included those involved in direct patient care (e.g. doctors, nurses, nursing assistants, physical/speech/respiratory therapists), and those assigned for non-patient care duties (e.g. environmental services, pharmacy, dietary, social work, case management). Eligible HCPs were invited to obtain free serologic testing between May 1 and June 30, 2020. This study was conducted under hospital operations jointly with approval for the use of a research serologic platform from the University of California Irvine IRB.

### Data collection

Demographic data and HCP job title/role were obtained from human resources records. Care of an infectious COVID-19 patient or patient room entry was identified using (1) electronic health record (EHR) records from start of the study period through 2 days before COVID-19 serology blood collection and (2) manager review of assigned duty locations for HCPs not identifiable through EHR records (e.g., environmental services) and (3) HCP interview for those in outbreaks. OH records provided assessment dates for COVID-19 symptoms and PCR results. Outbreak investigations were conducted by the Epidemiology and Infection Prevention Program (includes authors of this paper) according to CDC guidance consisting of investigation of every suspected or confirmed COVID-19 HCPs for exposure assessment and contact tracing for any linkages between cases by time, location, and activities. Any HCPs meeting exposure criteria were evaluated by Occupational Health, tested by COVID-19 PCR, monitored for symptoms, and furloughed as necessary. Any persons unexpectedly found to be COVID-19 positive were also evaluated for possible exposure to HCPs and other patients. In addition, HCP zipcodes were geocoded to obtain census-based socioeconomic status (SES) variables focusing on income and housing characteristics (Table 1) [12].

**Table 1:** Characteristics of healthcare professional participants by serologic status

Variable	All Participants	Seropositive	Seronegative	*p* value^a^
	N (%)	N (%)	N (%)	
Total N	654	87	567	
Invitation cohort criteria^b^				
Occupational health visit	273 (41.7)	52 (59.8)	166 (29.3)	< 0.001
Designated COVID-19 unit	275 (42.0)	31 (35.6)	244 (43.0)	0.19
Non-COVID-19 unit (control)	247 (37.8)	36 (41.4)	211 (37.2)	0.46
COVID-19 HCP-outbreak unit	272 (41.6)	46 (52.9)	226 (39.9)	0.02
COVID-19 non-HCP-outbreak unit	198 (30.3)	27 (31.0)	171 (30.2)	0.86
Age (mean, SD)	40	41 (47.1)	40 (7.1)	0.24
Male	181 (27.7)	27 (31.0)	154 (27.2)	0.42
Race^c^				
White	174 (26.6)	21 (24.1)	153 (27.0)	0.23
Asian	310 (47.4)	39 (44.8)	271 (47.8)	
Black	10 (1.5)	2 (2.3)	10 (1.8)	
Other	131 (20.0)	17 (19.5)	46 (8.1)	
Latino ethnicity	101 (15.4)	21 (24.1)	80 (14.1)	0.02
Socioeconomic status				
% Living in zipcodes where 10% or more have income below poverty line	189 (28.9)	27 (31.0)	162 (28.6)	0.64
% Living in zipcodes where 80% or more have income below median household income	237 (36.2)	38 (43.7)	199 (35.1)	0.12
% Living in zipcodes where owner-occupied household is less than 50%	350 (53.5)	54 (62.1)	296 (52.2)	0.09
% Living in zipcodes where 25% or more live in a house with 1.5 or more occupants per room	312 (47.7)	52 (59.8)	260 (45.9)	0.02
% Living in zipcodes where 50% households live in structures containing ≥ 5 units	307 (46.9)	42 (48.3)	265 (46.7)	0.79
Comorbidities (Any)	141 (21.6)	21 (24.1)	120 (21.2)	0.53
HCP role				
Registered nurse	382 (58.4)	53 (60.9)	329 (58.0)	0.12
Nurse aide	47 (7.2)	12 (13.8)	35 (6.2)	
Physical/occupational therapist	12 (1.8)	2 (2.3)	10 (1.8)	
Respiratory therapist	8 (1.2)	1 (1.1)	7 (1.2)	
Physician	99 (15.1)	7 (8.0)	92 (16.2)	
Environmental services	12 (1.8)	1 (1.1)	11 (1.9)	
Other-direct patient care	33 (5.0)	2 (2.3)	31 (5.5)	
Not direct Pt care	61 (9.3)	9 (10.3)	52 (9.2)	
COVID-19 patient care (Any)—Ehr review^d^	276 (42.2)	35 (40.2)	241 (42.5)	0.69
COVID-19 patient care—self-report^c^	355 (54.3)	41 (47.1)	314 (55.4)	0.35
Aerosol generating procedure^c^	97 (14.8)	8 (9.2)	89 (15.7)	0.11
Works in ICU^c^	153 (23.4)	19 (21.8)	134 (23.6)	0.71
PCR results				
Positive	54 (8.3)	41 (47.1)	13 (2.3)	< 0.01
Negative	245 (37.5)	19 (21.8)	226 (39.9)	
Unknown	355 (54.3)	27 (31.0)	328 (57.8)	
Any COVID-19 symptom on self-report^c^	395 (60.4)	62 (71.3)	333 (58.7)	< 0.01
No symptom reported	230 (35.2)	17 (19.5)	213 (37.6)	
Symptom type				
Non febrile illness	222 (33.9)	19 (21.8)	203 (35.8)	0.01
Fever	173 (26.5)	43 (49.4)	130 (22.9)	< 0.01
Fatigue	234 (35.8)	49 (56.3)	185 (32.6)	< 0.01
Chills	172 (26.3)	46 (52.9)	126 (22.2)	< 0.01
Myalgia	197 (30.1)	47 (54.0)	150 (26.5)	< 0.01
Congestion	298 (45.6)	42 (48.3)	256 (45.1)	0.30
Cough	224 (34.3)	37 (42.5)	187 (33.0)	0.03
Loss of smell	58 (8.9)	30 (34.5)	28 (4.9)	< 0.01
Shortness of breath	106 (16.2)	21 (24.1)	85 (15.0)	0.02
Days between symptoms and serology sample collection				
< 14 days	30 (4.6)	4 (4.6)	26 (4.6)	0.74
15–29 days	58 (8.9)	10 (11.5)	48 (8.5)	0.69
30–44 days	17 (2.6)	7 (8.0)	10 (1.8)	< 0.01
45–59 days	107 (16.4)	23 (26.4)	84 (14.8)	0.04
60–74 days	13 (2.0)	2 (2.3)	11 (1.9)	0.67
≥ 75 days	169 (25.8)	15 (17.2)	154 (27.2)	< 0.01

^a^*p* value = chi square comparing seropositive with seronegative

^b^Participants assigned to cohort if met criteria at any time during study period and prior to serology sample collection. Occupational Health Visit = visited occupational health; Designated COVID-19 Unit = HCP (healthcare professional) assigned to work in designated COVID-19 unit. Non-COVID-19 Unit (Control) = HCP assigned to work in units that did not admit COVID-19 patients. COVID-19 HCP Outbreak Unit = HCP assigned to work in unit where there was an ongoing HCP outbreak of COVID-19. COVID-19 Non-HCP Outbreak Unit = HCP assigned to work in a unit where there was not an active HCP COVID-19 outbreak

^c^Self-reported on REDCAP survey

^d^COVID-19 patient care confirmed by Electronic Health Record (EHR)

Eligible HCPs also completed a REDCAP survey requesting information on demographics, residential zipcode, comorbidities, COVID-19 patient-care (including aerosol generating procedures (AGPs)), and COVID-19 symptom history (including type/time of onset). Community COVID-19 prevalence was obtained through the Orange County Health Care Agency [13].

### Laboratory testing

To optimize capture of seropositive HCPs, serology was assessed using two platforms, a novel, high-sensitivity Fingerstick Coronavirus Antigen Microarray (COVAM) measuring IgG/IgM antibodies against 67 respiratory viruses, including 11 SARS-CoV-2 antigens, and a Food and Drug Administration Emergency Use Authorized serology assay (FDA-EUA) [14–17]. The primary FDA-EUA assay was Diazyme SARS-CoV-2 IgGdetecting antibodies against SARS-CoV-2 nucleocapsid(N) and spike(S) proteins [17]. To address potential performance characteristic variability, results were reflexed for confirmation by an alternate FDA-EUA assay (Beckman Access SARS-CoV-2 IgG assay against S protein or Abbott Architect SARS-CoV-2 IgG assay against N protein) when Diazyme results were non-reactive in PCR-confirmed or clinically suspected prior SARS-CoV-2 infection [17–20]. COVID-19 RT-PCR testing was performed using DiaSorin Molecular Simplexa, m2000 RealTime or Xpert Xpress SARS-CoV-2 [21–23]. PCR results from other facilities were obtained from OH records.

### Analysis

Percent participation was calculated among each invited cohort. Demographics and characteristics were evaluated as a proportion of participants. Multivariable logistic regression models evaluated the impact of the following variables on the composite outcome of COVID-19 infection defined as either seropositivity or PCR-positivity: demographics, HCP role, comorbidities, COVID-19 patient care (separately assessed by EHR documentation and survey report), ICU assignment, COVID-19 unit assignment, assignment in a unit during an active HCP outbreak, and geocoded SES variables. Self-reported AGP was collinear with COVID-19 care unit and ICU variables, and hence not included in this model. SES variables were evaluated according to previously published literature where possible, including: percent living in zipcodes where ≥ 10% have income below the poverty line, where ≥ 80% have income below the median household income, where ≥ 25% live in a house with 1.5 or more occupants/room, and where 50% households live in structures containing ≥ 5 units [24, 25]. We assessed the association between Latino HCPs and living in areas with higher percentages of Latino residents (above median, 35%) to assess whether ethnicity can be considered a community-level exposure risk. Percent owner-occupied housing was evaluated using the median cut-point (58%) of the dataset since owner occupancy in our county is higher than the national average and variable due to large wealth gradients.

Separately, we evaluated symptom association with seropositivity, creating symptom groups using correlation matrices that were then assessed for association with seropositivity using multivariable logistic regression. Finally, performance of each serologic assay was compared.

## Results

A total of 1,320 HCPs were invited, including 476 seen by OH, 494 assigned to a designated COVID-19 unit, 388 assigned to a unit that experienced a COVID-19 HCP-outbreak, and 378 assigned to a matched control unit (non-COVID unit and without HCP-outbreak). Some HCPs are counted in multiple categories (e.g. seen by OH and worked in an HCP-outbreak unit). Among all invited, 654 HCPs participated in serologic testing (623 completed surveys). Participation was similar between cohorts: OH invited cohort (57.4% (273/476)), COVID-19-designated unit (55.7% (275/494), COVID-19 HCP-outbreak unit (50.7% 197/388), and matched non-COVID-19, non-HCP-outbreak control unit (50.0% (198/378). Table 1 shows cohort subsets and participant characteristics. Compared to all invited HCPs, participants with prior COVID-19 PCR-positivity were more likely to participate (8.3%, 54/654 versus 7.0%, 92/1320), though not statistically significant, *p* = 0.35. A total 87 (13.3%) HCPs were seropositive for COVID-19 by either serology assay. Fourteen specimen testing negativeby FDA-EUA were reflexed to alternate FDA-EUA testing, with 2 reactive results. Countywide test-positivity during the study period was 7.2% (1651/22,882).

Bivariate evaluation showed similar demographics, comorbidities, HCP role, COVID-19 patient-care, AGP performance, and assignment to ICU and COVID-19-designated units between seropositive and seronegative participants (Table 1). Seropositivity was higher among HCPs seen by OH, prior symptomatic COVID-19, assigned to a HCP-outbreak unit, of Latino ethnicity, and living in a zipcode where ≥ 25% of residents live in households with more than 1.5 occupants/room (Table 1). Notably, 60.8% (N = 52) of seropositive HCP never cared for a COVID-19 patient based upon EHR documentation (52.9% (N = 46) based upon survey self-report).

The majority (62/87, 71.3%) of seropositive HCPs reported at least one COVID-19 symptom preceding sero-testing; most (47/87, 54.0%) had symptoms ≥ 30 days before testing. Anosmia occurred in 34.5% (N = 30) of seropositives compared to 4.9% (N = 28) of seronegatives.. All PCR-positive participants were seropositive. Latino HCPs were more likely to live in areas with higher Latino populations (64%, N = 63/98) compared to non-Latino HCPs (44%, N = 238/542, *p* < 0.01; Additional file 1: Table A).

Multivariable regression identified male gender (OR 1.79, CI 1.05–3.04, *p* = 0.03), Latino ethnicity (OR 2.10, CI 1.12–3.96, *p* = 0.02), residence in a community with low owner-occupied housing (OR = 1.63, CI = 1.00–2.64, *p* = 0.05), and working in a unit with an HCP outbreak (OR 2.21, CI 1.28–3.81, *p* < 0.01) as significantly associated with COVID-19 infection (sero- or PCR-positive) after adjusting for HCP role, comorbidities, and documented COVID-19 patient care (Table 2). Working in a COVID-19 unit was associated with a lower likelihood of COVID-19 (OR 0.53, CI = 0.30–0.94, *p* = 0.03).

**Table 2:** Multivariate regression model evaluating epidemiologic risk factors for positive serology or PCR (composite)

Variable	OR (CI)	*p* value
Age^a^ (years)		0.40
< 35	1.00	
35 to < 50	1.53 (0.88–2.69)	
50 to < 60	1.68 (0.82–3.45)	
> 60	1.42 (0.45–4.44)	
Male	1.79 (1.04–3.94)	0.03
HCP role^a^		0.35
Registered nurse	1.29 (0.57–2.95)	
Nurse aide	1.02 (0.20–5.74)	
Physical/occupational therapist	1.05 (0.12–9.36)	
Respiratory therapist	0.47 (0.20–1.13)	
Physician	0.32 (0.07–1.43)	
Environmental services	0.19 (0.02–1.69)	
Other-direct patient care	0.95 (0.41–2.19)	
Not direct Pt care	1.29 (0.57–2.95)	
Comorbidities (Any)	1.01 (0.56–1.79)	0.98
COVID patient care by EHR^b^	1.10 (0.64–1.88)	0.73
Latino ethnicity^c^	2.1 (1.12–3.96)	0.02
% Living in zipcode where < 58% (median) live in owner-occupied home	1.63 (1.00–2.64)	0.05
Intensive care unit	0.75 (0.41–1.37)	0.34
HCP COVID outbreak unit	2.21 (1.28–3.81)	< 0.01
Designated COVID care unit	0.53 (0.30–0.94)	0.03

^a^Referents for categorical variables: age ≤ 35 years, HCP Role = Registered nurse

^b^EHR = Electronic Health Record. Note self-reported aerosol generating procedure was collinear with COVID care unit and ICU variables and were not included in this model

Highly correlated symptoms were (*rho* > 0.50) were grouped together: (1) fever, chills, myalgias and (2) cough/congestion. When adjusting for age, gender, ethnicity, and comorbidities, fevers/chills/myalgias (OR 2.00, CI 1.03–3.90, *p* = 0.04) and anosmia (OR 8.81, CI 4.44–17.50, *p* < 0.0001) were associated with COVID-19 (Table 3).

**Table 3:** Multivariate regression model evaluating clinical characteristics associated with positive serology or PCR (composite)

Variable	OR (CI)	*p* value
Age^a^ (years)		0.13
< 35	1.00	
35 to < 50	1.71 (0.91–3.27)	
50 to < 60	2.47 (1.13–5.41)	
> 60	1.57 (0.42–5.86)	
Male	1.48 (0.84–2.61)	0.18
Latino ethnicity	2.06 (1.09–3.87)	0.03
Comorbidities (any)	0.87 (0.45–1.69)	0.69
Fever/chills/myalgias^b^	2.00 (1.02–3.98)	0.04
Cough/congestion^b^	1.08 (0.55–2.39)	0.83
Loss of smell	8.81 (4.43–17.59)	< 0.01
Shortness of breath	0.88 (0.44–1.74)	0.70

^a^Referent for age ≤ 35 years

^b^Symptoms highly correlated (> 0.50) with each other and evaluated as a composite

The COVAM assay identified 25 additional seropositive individuals (78 [12%]) compared to FDA-EUA assay (53 [8%])) (Table 4), *p* < 0.01. The FDA-EUA assay had a higher (statistically insignificant) proportion of seropositivesreporting any COVID-19 symptoms (fever, chills, myalgias, fatigue, anosmia, cough, and shortness of breath), while COVAM had a higher proportion that were asymptomatic, had only non-febrile illness, or symptoms > 75 days before testing. When restricting to 41 PCR-confirmed participants (Table 5), all were seropositive by COVAM assay while 38 (92.7%) were seropositive by the FDA-EUA assay; among the two additional patients detected by COVAM, both had symptoms > 60 days before blood draw.

**Table 4:** Comparative evaluation of FDA-EUA versus COVAM serology by symptom characteristics

	Symptom characteristics by serology assay				Percent seropositive within each symptom category	
	FDA-EUA assay or microarray	N (%) of FDA-EUA serology with characteristic	N (%) of COVAM serology with characteristic	*p* value	N (%) of characteristic with positive FDA-EUA serology	N (%) of characteristic with positive COVAM serology
Seropositive—total	87	53	78	< 0.01	60.9%	89.7%
Reported to occupational health	67 (77.0)	46 (86.8)	63 (80.8)	NS	68.7%	94.0%
Any symptom reported	62 (71.3)	42 (79.2)	58 (74.4)	NS	67.7%	93.5%
No symptom reported	25 (28.7)	11 (20.8)	20 (25.6)	NS	44.0%	80.0%
Symptom type						
Non febrile illness	19 (21.8)	8 (15.1)	16 (20.5)	NS	42.1%	84.2%
Fever	43 (49.4)	34 (64.2)	42 (53.8)	NS	79.1%	97.7%
Fatigue	49 (56.3)	35 (66.0)	46 (59.0)	NS	71.4%	93.9%
Chills	46 (52.9)	34 (64.2)	43 (55.1)	NS	73.9%	93.5%
Myalgia	47 (54.0)	33 (62.3)	45 (57.7)	NS	70.2%	95.7%
Congestion	42 (48.3)	25 (47.2)	39 (50.0)	NS	59.5%	92.9%
Cough	37 (42.5)	28 (52.8)	34 (43.6)	NS	75.7%	91.9%
Loss of smell	30 (34.5)	28 (52.8)	30 (38.5)	NS	93.3%	100.0%
Shortness of breath	21 (24.1)	16 (30.2)	19 (24.4)	NS	76.2%	96.3%
Days between symptoms and serology sample collection				NS		
< 14 days	4 (4.6)	3 (5.7)	4 (5.1)		75.0%	100.0%
15–29 days	10 (11.5)	8 (15.1)	9 (11.5)		80.0%	90.0%
30–44 days	7 (8.0)	7 (13.2)	7 (9.0)		100.0%	100.0%
45–59 days	23 (26.4)	19 (35.8)	23 (29.5)		82.6%	100.0%
60–74 days	2 (2.3)	1 (1.9)	2 (2.6)		50.0%	100.0%
≥ 75 days	15 (17.2)	4 (7.5)	12 (15.4)		26.7%	80.0%

FDA-EUA, Food and drug administration emergency use authorization; COVAM, coronavirus antigen microarray

^a^*p* value = chi square comparing FDA-EUA with COVAM serology

**Table 5:** Comparison of FDA-EUA and COVAM seropositivity and symptom characteristics among those with PCR-confirmed COVID

Seropositive and PCR-positives	FDA-EUA Assay or COVAM N (%)		FDA-EUA assay serology N (%)		COVAM serology N (%)		*p* value^a^
PCR-positive—total	41		38		41		
Seropositive	41		38		41		
Reported to Occ health	41		38		41		
No symptom reported	4	9.8%	4	10.5%	4	9.8%	0.81
Any symptom reported	37	90.2%	34	89.5%	37	90.2%	0.81
Symptom type							
Fever	31	75.6%	30	78.9%	31	75.6%	0.77
Non febrile illness	6	14.6%	4	10.5%	6	14.6%	0.84
Fatigue	31	75.6%	29	76.3%	31	75.6%	0.89
Chills	30	73.2%	28	73.7%	30	73.2%	0.90
Myalgia	30	73.2%	28	73.7%	30	73.2%	0.90
Congestion	21	51.2%	19	50.0%	21	51.2%	0.94
Cough	24	58.5%	23	60.5%	24	58.5%	0.91
Loss of smell	27	65.9%	26	68.4%	27	65.9%	0.87
Shortness of breath	15	36.6%	14	36.8%	15	36.6%	0.93
Days between symptoms and serology sample collection							
< 14 days	4	9.8%	3	7.9%	4	9.8%	0.96
15–29 days	7	17.1%	6	15.8%	7	17.1%	
30–44 days	7	17.1%	7	18.4%	7	17.1%	
45–59 days	17	41.5%	16	42.1%	17	41.5%	
60–74 days	1	2.4%	1	2.6%	1	2.4%	
≥ 75 days	1	2.4%	1	2.6%	1	2.4%	

FDA-EUA, Food and drug administration emergency use authorization; COVAM, coronavirus antigen microarray

^a^*p* value = chi square comparing FDA-EUA with COVAM serology

During the study period, which occurred before universal masking and mandatory N95 use, there were three units with HCP-outbreaks involving 18 HCPs; of these, 8 (44.4%) were exposed to an ill coworker, 6 (33.3%) had no known exposure source, and 3 (16.7%) had community exposure source. Only 1 (5.6%) HCP infection was plausibly related to patient exposure due to breach of personal protective equipment. The first began with an HCP who traveled to an area with widespread COVID-19 and had never cared for a COVID-19 patient; this HCP developed myalgias/arthralgiaswhile at work and sore throat the following day, prompting symptom report and testing. While symptomatic, the HCP interacted directly with two other HCPs who subsequently developed COVID-19 within 4–5 days. Their interactions involved hand-off of a nursing cell phone and sharing lunch in a breakroom.

The second outbreak began with a HCP who had no clear source for COVID-19 at work or in the community. The HCP developed symptoms while working and likely infected three other coworkers who worked the same unit and shift; these HCPs subsequently developed symptoms while working, resulting in a cascade of four additional COVID-19 infections in HCPs working during the same shift and/or shared spaces (e.g., breakroom, nursing station, skills class). One physician who did not regularly work on the unit spent less than 1 h at the nursing station and developed COVID-19 without having entered a COVID-19 patient room.

In the third outbreak, a potluck led to 6 HCPs developing COVID-19. Preceding the potluck, a patient tested positive for COVID-19 after being unrecognized while admitted. This patient underwent emergent resuscitation and intubation before COVID-19 diagnosis, but none of the code blue providers developed COVID-19. Five of the six HCPs had not provided care for a COVID-19 patient in the weeks prior to developing symptoms. An administrative staff that assisted with obtaining supplies during the code blue who did not have direct patient contact most likely acquired illness from symptomatic coworkers in the nursing station based on exposure history.

## Discussion

Appropriate attribution of true HCP exposure risk must be contextualized by exposure sources faced in community settings and non-patient care work activities, while simultaneously accounting for the infection prevention strategies in place within healthcare settings to mitigate patient exposures. The top predictors of COVID-19 seropositivity were working in an HCP-outbreak unit, Latino ethnicity, and living in zip code with lower owner-occupied housing, reflecting the important contribution of coworkers and community exposures above and beyond documented COVID-19 patient care. In fact, we found that working in a COVID-19 unit (with contact, eye, and droplet-based mask precautions) was protective against infection, after accounting for the above community and work-related factors, including HCP role and documented care of a COVID-19 patient, suggesting that infection prevention protocols and practices are highly effective in preventing patient-to-HCP transmission.

The majority of HCP workhours are spent performing indirect patient-care tasks, such as charting, rounding, or discussing/coordinating care. Direct patient-facing care constitutes 20–40% of HCP time, which decreases by 18% in contact precautions rooms [26–28]. During a pandemic, heightened awareness of personal risk results in high PPE and hand hygiene compliance, further decreasing the likelihood of patient care exposures [29–32]. HCPs spend comparatively more time in communal settings, often in confined and shared spaces such as nursing stations, physician workrooms, breakrooms, and conference rooms [33, 34]. The propensity to work while ill further exacerbates coworker exposure risk, an unintended consequence of strong work ethics that lead to working long hours despite physical discomfort or sickness [35].

Our findings that 60% of HCPs involved in COVID-19 outbreaks never cared for a COVID-19 patient and that HCP infections propagated between coworkers highlights the importance of robust daily symptom screening, enforcement of working-well policies, and strict compliance with universal masking and social distancing in communal spaces. Current regulatory agencies emphasize high standards for hand hygiene, PPE compliance, and environmental cleaning practices for patient safety; translating these standards to shared HCP spaces and activities is imperative, particularly during a pandemic [36, 37]. This includes increasing the number and strategic placement of hand hygiene stations, environmental cleaning products for high touch items in workstations and breakrooms, workflow and structural modifications to minimize crowding, and robust enforcement of protocol compliance.

County COVID-19 cases were higher among Latino and densely populated communities, mimicking national trends showing disproportionately greater burden in socioeconomically disadvantaged areas [13, 24]. COVID-19 exposures are more likely in communities with household crowding, low-wage essential workers, and reliance on public transportation, increasing the likelihood of encountering crowded conditions [24, 38]. This has two important implications for hospital pandemic response. First, infection prevention strategies to reduce HCP risk should also address community level risks within the workforce, targeting outreach to HCPs from high risk communities, providing culturally and linguistically appropriate education on how to minimize risks within both healthcare and community settings. Since HCPs in socioeconomically disadvantaged communities live with household members who also carry heightened risk for acquiring infection, assuring HCPs are aware of home-based infection prevention practices may afford additional protection. Second, essential workplaces must partner with public health to educate, support contact tracing and quarantining strategies, and facilitate timely testing and management in high-risk communities where their workers reside.

HCP risk perception is integral to promoting behaviors that reduce exposures. Fear and concern about acquiring COVID-19 results in high adherence to hand hygiene and PPE during patient interactions [30]. In contrast, HCPs perceive coworkers or community exposures as less risky, which can increase transmission opportunities in non-clinical spaces (e.g., breakrooms). The earliest reports of high COVID-19 risk among HCPs occurred during inadequate PPE and nascent infection prevention protocols [1, 3, 30]. Subsequently, national and state regulatory bodies, nursing unions, and the media assumed inadequate PPE was the primary exposure risk in healthcare settings, missing important contributions from coworker or community risk. This led to increasingly intensive direct-care PPE requirements without equal attention to contributions from coworker and community exposures. Notably, the outbreaks in this study occurred before N95 requirements for patient care and we found that droplet-based PPE successfully prevented COVID transmission from patients-to-HCP, while coworker-to-coworker exposures were the sources of unit HCP outbreaks.

This study allowed comparison between FDA-EUA serologic assays and a novel microarray assay capable of differentiating 67 respiratory virus antigen, including influenza and four common cold coronaviruses [15]. Though we found high concordance between the two assays, the microarray identified additional individuals with asymptomatic or non-febrile infection, or illness beyond 75 days before testing. The microarray assay could be particularly helpful during the cold and flu season given its ability to differentiate between COVID-19 and other respiratory viruses.

Our findings are limited by a single institutional experience. Second, our county has an overall higher per-capita income compared to other counties although the wealth gradient across the county is notable and able to identify SES-based risk factors. Third, participation was voluntary which could introduce sampling bias, although participation across the invited cohorts were similar.

## Conclusions

HCP COVID-19 exposure risks must be evaluated and interpreted within the full context of workplace and community exposure sources. When accounting for the protections in place for direct patient-care activities, workplace and community exposures appear to dominate COVID-19 risk. Additional investments are needed to account for familiar behaviors and shared meals among co-workers and improve infection prevention strategies to reduce transmission. In addition, investments are needed to target pandemic response efforts to lower socioeconomic status communities where essential workers live. Healthcare systems should consider opportunities to partner with public health to leverage COVID-prevention expertise to these areas.

## Supplementary Information

**Additional file 1**. Supplemental Table A: This table shows the proportion of healthcare professionals who are Latino vs Non-Latino, stratified by the percentage of Latino residents in the communities in which they live. These data demonstrate that Latino healthcare workers live in areas where Latino population is higher.

## Data Availability

All data are available to authors and subject to health information protections.
