# Supplementary material for: Infection prevention strategies are highly protective in COVID-19 units while main risks to healthcare professionals come from coworkers and the community
Source: Antimicrob Resist Infect Control. 2021 Nov 22;10:163. doi: 10.1186/s13756-021-01031-5 (PMC8608236; doi:10.1186/s13756-021-01031-5)
Supplement: Supplementary file 1 — Additional file 1. Supplemental Table A: This table shows the proportion of healthcare professionals who are Latino vs Non-Latino, stratified by the percentage of Latino residents in the communities in which they live. These data demonstrate that Latino healthcare workers live in areas where Latino population is higher. [file 13756_2021_1031_MOESM1_ESM.docx]

**Supplemental Table A: Proportion of Latino vs Non-Latino Healthcare Professionals Stratified by Community Percent of Latino Residents**

|  | **Latino N (%)** | **Non-Latino N (%)** | **P-value**^1^ |
| --- | --- | --- | --- |
| **Number of Healthcare Professionals (HCPs)** | 98 | 542 |  |
| **Living in zip code >= 35% (median) Latino residents** | 63 (64) | 238 (44) | <0.01 |
| **Living in zip code < 35% (median) Latino residents** | 35 (36) | 304 (56) |  |
| **Living in zip code >= 17% (25^th^ percentile) Latino residents** | 84 (86) | 401 (74) | 0.01 |
| **Living in zip code >= 47% (75^th^ percentile) Latino residents** | 49 (50) | 128 (24) | < 0.01 |

^1^P-value = chi square comparing proportion of Latino versus non-Latino HCPs.
